# 3-[4-(2-Chloro­benzyl­idene­amino)-3-methyl-5-sulfanyl­idene-4,5-dihydro-1*H*-1,2,4-triazol-1-yl]-1,3-diphenyl­propan-1-one

**DOI:** 10.1107/S1600536811032983

**Published:** 2011-08-27

**Authors:** Wei Wang, Qing-lei Liu, Yan Gao, Wen-peng Wu, Chao Xu

**Affiliations:** a School of Perfume and Aroma Technology, Shanghai Institute of Technology, Shanghai 200235, People’s Republic of China; b School of Chemical Engineering, University of Science and Technology LiaoNing, Anshan 114051, People’s Republic of China

## Abstract

In the title mol­ecule, C_25_H_21_ClN_4_OS, the triazole ring forms dihedral angles of 47.9 (2), 84.5 (2) and 3.9 (2)° with the two phenyl rings and the chloro­phenyl ring, respectively. The chloro­phenyl ring, the triazole ring and the conjugative linker between the two aromatic rings are nearly coplanar with an r.m.s. deviation of 0.0483 (2) Å and a maximum deviation of 0.0911 (2) Å.

## Related literature

For the structures of related 1,2,4-triazole-5(4*H*)-thione derivatives, see: Al-Tamimi *et al.* (2010 ▶); Fun *et al.* (2009 ▶); Gao *et al.* (2011 ▶); Tan *et al.* (2010 ▶); Wang *et al.* (2011 ▶); Zhao *et al.* (2010 ▶).
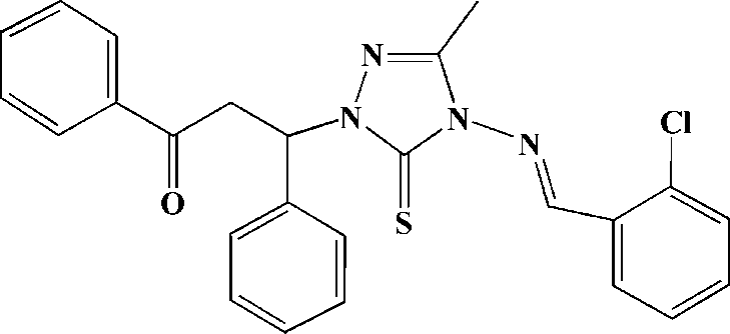


## Experimental

### 

#### Crystal data

C_25_H_21_ClN_4_OS
*M* *_r_* = 460.97
Triclinic, 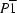

*a* = 8.3347 (9) Å
*b* = 10.6029 (12) Å
*c* = 13.4262 (16) Å
α = 87.907 (19)°
β = 81.026 (18)°
γ = 82.024 (17)°
*V* = 1160.5 (2) Å^3^
*Z* = 2
Mo *K*α radiation
μ = 0.28 mm^−1^
*T* = 113 K
0.18 × 0.16 × 0.10 mm

#### Data collection

Rigaku Saturn CCD area-detector diffractometer
Absorption correction: multi-scan (*CrystalClear*; Rigaku/MSC, 2005 ▶) *T*_min_ = 0.951, *T*_max_ = 0.973
14988 measured reflections
5478 independent reflections
3741 reflections with *I* > 2σ(*I*)
*R*_int_ = 0.047

#### Refinement

*R*[*F* ^2^ > 2σ(*F* ^2^)] = 0.055
*wR*(*F* ^2^) = 0.127
*S* = 1.02
5478 reflections
290 parameters
H-atom parameters constrained
Δρ_max_ = 0.23 e Å^−3^
Δρ_min_ = −0.35 e Å^−3^

### 

Data collection: *CrystalClear* (Rigaku/MSC, 2005 ▶); cell refinement: *CrystalClear*; data reduction: *CrystalClear*; program(s) used to solve structure: *SHELXS97* (Sheldrick, 2008 ▶); program(s) used to refine structure: *SHELXL97* (Sheldrick, 2008 ▶); molecular graphics: *SHELXTL* (Sheldrick, 2008 ▶); software used to prepare material for publication: *SHELXTL*.

## Supplementary Material

Crystal structure: contains datablock(s) global, I. DOI: 10.1107/S1600536811032983/zk2023sup1.cif

Structure factors: contains datablock(s) I. DOI: 10.1107/S1600536811032983/zk2023Isup2.hkl

Supplementary material file. DOI: 10.1107/S1600536811032983/zk2023Isup3.cml

Additional supplementary materials:  crystallographic information; 3D view; checkCIF report

## Figures and Tables

**Fig. 1.**
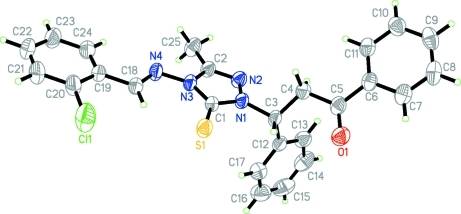
View of the molecule of (I) showing the atom-labelling scheme. Displacement ellipsoids are drawn at the 60% probability level.

## supplementary crystallographic information

### Comment

In continuation of structural study of Mannich bases derivatives synthesized by reactions of the amino heterocycles and aromatic aldehydes in our group(Wang *et al.*, 2011), we present here the crystal structure of the title compound, (I).

The bond lengths and angles in compound (I) are found to have normal values comparable with those reported in the related 1,2,4-triazole- 5(4*H*)-thione derivatives (Al-Tamimi *et al.*, 1987; Fun *et al.* (2009); Tan *et al.* (2010); Wang *et al.*, 2011;). The C1 and C2 atoms in the 1,2,4-triazole ring show distorted C*_sp_*^2^ hybridization states with the bond angles of 101.84 (17)° (N1—C1—N3), 130.42 (15)°(N3—C1—S1), 110.66 (18)°(N2—C2—N3) and 126.39 (19) °(N2—C2—C25), which are similar to the reported triazole derivatives (Zhao *et al.*, 2010; Gao *et al.*, 2011). The 1,2,4-triazole ring forms the dihedral angles of 47.9 (2), 84.5 (2) and 3.9 (2)° with two phenyl rings and chlorophenyl rings, respectively. Three parts of chlorophenyl ring, triazole ring and the conjugative linker between this two armotic rings are nearly coplanar with an r.m.s. derivation of 0.0483 (2)Å and maximum deviation of 0.0911 (2) Å. The values of torsion angles N3—N4—C18—C19 [179.87 (17)°], N4—C18—C19—C20 [173.6 (2)°] and C2—N3—N4—C18 [173.88 (19)°] also confirm this results.

### Experimental

The title compound was synthesized with the reaction of 2-chlorobenzaldehyde(2.0 mmol) and 3-(4-amino-3-methyl-5-thioxo-4,5- dihydro-1*H*-1,2,4-triazol-1-yl)-1,3-diphenylpropan-1-one(2.0 mmol) by refluxing in ethnol. The reaction progress was monitored *via* TLC. The resulting precipitate was filtered off, washed with cold ethanol, dried and purified to give the target product as colorless solid in 78% yield. Crystals of (I) suitable for single-crystal X-ray analysis were grown by slow evaporation of a solution in chloroform-ethanol (1:1).

### Refinement

H atoms were positioned geometrically and refined as riding (C—H = 0.95–1.00 Å) and allowed to ride on their parent atoms, with *U*_iso_(H) = 1.2*U*_eq_(parent) or 1.5*U*_eq_(parent).

### Crystal data

**Table:**

C_25_H_21_ClN_4_OS	*Z* = 2
*M**_r_* = 460.97	*F*(000) = 480
Triclinic, *P*1	*D*_x_ = 1.319 Mg m^−^^3^
Hall symbol: -P 1	Mo *K*α radiation, λ = 0.71073 Å
*a* = 8.3347 (9) Å	Cell parameters from 3699 reflections
*b* = 10.6029 (12) Å	θ = 1.5–27.9°
*c* = 13.4262 (16) Å	µ = 0.28 mm^−^^1^
α = 87.907 (19)°	*T* = 113 K
β = 81.026 (18)°	Prism, colorless
γ = 82.024 (17)°	0.18 × 0.16 × 0.10 mm
*V* = 1160.5 (2) Å^3^	

### Data collection

**Table:**

Rigaku Saturn CCD area-detector diffractometer	5478 independent reflections
Radiation source: rotating anode	3741 reflections with *I* > 2σ(*I*)
multilayer	*R*_int_ = 0.047
Detector resolution: 14.63 pixels mm^-1^	θ_max_ = 27.8°, θ_min_ = 1.5°
φ and ω scans	*h* = −10→10
Absorption correction: multi-scan (*CrystalClear*; Rigaku/MSC, 2005)	*k* = −13→12
*T*_min_ = 0.951, *T*_max_ = 0.973	*l* = −17→17
14988 measured reflections	

### Refinement

**Table:**

Refinement on *F*^2^	Primary atom site location: structure-invariant direct methods
Least-squares matrix: full	Secondary atom site location: difference Fourier map
*R*[*F*^2^ > 2σ(*F*^2^)] = 0.055	Hydrogen site location: inferred from neighbouring sites
*wR*(*F*^2^) = 0.127	H-atom parameters constrained
*S* = 1.02	*w* = 1/[σ^2^(*F*_o_^2^) + (0.0498*P*)^2^] where *P* = (*F*_o_^2^ + 2*F*_c_^2^)/3
5478 reflections	(Δ/σ)_max_ < 0.001
290 parameters	Δρ_max_ = 0.23 e Å^−^^3^
0 restraints	Δρ_min_ = −0.35 e Å^−^^3^

### Special details

**Table:**

Geometry. All e.s.d.'s (except the e.s.d. in the dihedral angle between two l.s. planes) are estimated using the full covariance matrix. The cell e.s.d.'s are taken into account individually in the estimation of e.s.d.'s in distances, angles and torsion angles; correlations between e.s.d.'s in cell parameters are only used when they are defined by crystal symmetry. An approximate (isotropic) treatment of cell e.s.d.'s is used for estimating e.s.d.'s involving l.s. planes.
Refinement. Refinement of *F*^2^ against ALL reflections. The weighted *R*-factor *wR* and goodness of fit *S* are based on *F*^2^, conventional *R*-factors *R* are based on *F*, with *F* set to zero for negative *F*^2^. The threshold expression of *F*^2^ > σ(*F*^2^) is used only for calculating *R*-factors(gt) *etc*. and is not relevant to the choice of reflections for refinement. *R*-factors based on *F*^2^ are statistically about twice as large as those based on *F*, and *R*- factors based on ALL data will be even larger.

### Fractional atomic coordinates and isotropic or equivalent isotropic displacement parameters (Å^2^)

**Table:**

	*x*	*y*	*z*	*U*_iso_*/*U*_eq_	
S1	0.29774 (8)	0.61784 (5)	0.10454 (4)	0.04087 (18)	
Cl1	0.20199 (11)	0.93846 (6)	−0.11093 (5)	0.0681 (3)	
O1	0.3085 (2)	0.22648 (14)	0.34675 (11)	0.0456 (4)	
N1	0.4144 (2)	0.59915 (15)	0.28411 (12)	0.0313 (4)	
N2	0.4657 (2)	0.66988 (15)	0.35541 (12)	0.0340 (4)	
N3	0.3976 (2)	0.79060 (15)	0.22639 (12)	0.0309 (4)	
N4	0.3676 (2)	0.90997 (15)	0.18009 (13)	0.0340 (4)	
C1	0.3707 (3)	0.66863 (19)	0.20309 (15)	0.0309 (5)	
C2	0.4535 (3)	0.78623 (19)	0.31822 (15)	0.0318 (5)	
C3	0.4152 (3)	0.46078 (18)	0.30128 (15)	0.0313 (5)	
H3	0.3646	0.4275	0.2465	0.038*	
C4	0.3078 (3)	0.43869 (18)	0.40126 (15)	0.0353 (5)	
H4A	0.2043	0.4980	0.4053	0.042*	
H4B	0.3646	0.4583	0.4571	0.042*	
C5	0.2675 (3)	0.3027 (2)	0.41478 (16)	0.0352 (5)	
C6	0.1735 (3)	0.26640 (19)	0.51340 (16)	0.0332 (5)	
C7	0.1321 (3)	0.1420 (2)	0.52621 (17)	0.0414 (6)	
H7	0.1621	0.0838	0.4722	0.050*	
C8	0.0481 (3)	0.1038 (2)	0.61687 (19)	0.0459 (6)	
H8	0.0223	0.0193	0.6254	0.055*	
C9	0.0018 (3)	0.1891 (2)	0.69522 (18)	0.0445 (6)	
H9	−0.0565	0.1629	0.7573	0.053*	
C10	0.0399 (3)	0.3124 (2)	0.68346 (17)	0.0424 (6)	
H10	0.0071	0.3707	0.7372	0.051*	
C11	0.1257 (3)	0.3505 (2)	0.59356 (16)	0.0375 (5)	
H11	0.1525	0.4349	0.5862	0.045*	
C12	0.5930 (3)	0.39735 (18)	0.29147 (15)	0.0327 (5)	
C13	0.6642 (3)	0.34362 (19)	0.37317 (17)	0.0400 (6)	
H13	0.6004	0.3426	0.4383	0.048*	
C14	0.8284 (3)	0.2916 (2)	0.3594 (2)	0.0516 (7)	
H14	0.8759	0.2543	0.4153	0.062*	
C15	0.9234 (3)	0.2933 (2)	0.2655 (2)	0.0551 (7)	
H15	1.0360	0.2586	0.2571	0.066*	
C16	0.8540 (3)	0.3458 (2)	0.1841 (2)	0.0543 (7)	
H16	0.9188	0.3470	0.1193	0.065*	
C17	0.6896 (3)	0.3970 (2)	0.19660 (17)	0.0429 (6)	
H17	0.6423	0.4322	0.1400	0.051*	
C18	0.3300 (3)	0.91863 (19)	0.09206 (15)	0.0341 (5)	
H18	0.3212	0.8449	0.0562	0.041*	
C19	0.3002 (3)	1.04705 (19)	0.04715 (15)	0.0318 (5)	
C20	0.2424 (3)	1.0664 (2)	−0.04535 (16)	0.0405 (6)	
C21	0.2152 (3)	1.1880 (2)	−0.08722 (18)	0.0469 (6)	
H21	0.1754	1.1999	−0.1499	0.056*	
C22	0.2463 (3)	1.2909 (2)	−0.03757 (18)	0.0473 (6)	
H22	0.2267	1.3740	−0.0658	0.057*	
C23	0.3057 (3)	1.2743 (2)	0.05313 (18)	0.0479 (6)	
H23	0.3284	1.3458	0.0865	0.057*	
C24	0.3322 (3)	1.1538 (2)	0.09534 (17)	0.0412 (6)	
H24	0.3728	1.1432	0.1579	0.049*	
C25	0.4944 (3)	0.9002 (2)	0.36488 (17)	0.0429 (6)	
H25A	0.5084	0.8804	0.4351	0.064*	
H25B	0.4054	0.9706	0.3628	0.064*	
H25C	0.5964	0.9248	0.3275	0.064*	

### Atomic displacement parameters (Å^2^)

**Table:**

	*U*^11^	*U*^22^	*U*^33^	*U*^12^	*U*^13^	*U*^23^
S1	0.0663 (4)	0.0293 (3)	0.0291 (3)	−0.0053 (3)	−0.0155 (3)	0.0028 (2)
Cl1	0.1153 (7)	0.0498 (4)	0.0527 (4)	−0.0258 (4)	−0.0429 (4)	0.0075 (3)
O1	0.0627 (12)	0.0358 (9)	0.0392 (9)	−0.0074 (8)	−0.0097 (8)	−0.0028 (7)
N1	0.0427 (11)	0.0253 (9)	0.0255 (9)	0.0001 (8)	−0.0093 (8)	0.0029 (7)
N2	0.0422 (11)	0.0294 (10)	0.0318 (10)	−0.0014 (8)	−0.0130 (8)	0.0007 (8)
N3	0.0379 (11)	0.0245 (9)	0.0297 (9)	−0.0008 (7)	−0.0074 (8)	0.0052 (7)
N4	0.0413 (11)	0.0263 (10)	0.0343 (10)	−0.0025 (8)	−0.0097 (8)	0.0074 (8)
C1	0.0385 (13)	0.0263 (11)	0.0258 (10)	0.0000 (9)	−0.0030 (9)	0.0036 (9)
C2	0.0366 (13)	0.0297 (12)	0.0300 (11)	0.0001 (9)	−0.0118 (9)	0.0014 (9)
C3	0.0427 (13)	0.0225 (11)	0.0287 (11)	−0.0015 (9)	−0.0087 (10)	0.0035 (8)
C4	0.0417 (14)	0.0294 (12)	0.0329 (12)	0.0003 (9)	−0.0056 (10)	0.0040 (9)
C5	0.0415 (14)	0.0311 (12)	0.0346 (12)	−0.0022 (10)	−0.0140 (10)	0.0053 (10)
C6	0.0352 (12)	0.0317 (12)	0.0344 (12)	−0.0033 (9)	−0.0133 (10)	0.0069 (9)
C7	0.0438 (14)	0.0366 (13)	0.0469 (14)	−0.0085 (10)	−0.0153 (11)	0.0054 (11)
C8	0.0456 (15)	0.0404 (14)	0.0563 (16)	−0.0148 (11)	−0.0179 (12)	0.0178 (12)
C9	0.0339 (13)	0.0569 (16)	0.0442 (14)	−0.0119 (11)	−0.0106 (11)	0.0193 (12)
C10	0.0413 (14)	0.0456 (14)	0.0389 (13)	−0.0035 (11)	−0.0061 (11)	0.0070 (11)
C11	0.0390 (14)	0.0346 (13)	0.0395 (13)	−0.0049 (10)	−0.0096 (10)	0.0070 (10)
C12	0.0417 (13)	0.0235 (11)	0.0324 (11)	−0.0001 (9)	−0.0088 (10)	0.0003 (9)
C13	0.0473 (15)	0.0352 (13)	0.0373 (12)	0.0020 (10)	−0.0122 (11)	−0.0012 (10)
C14	0.0530 (17)	0.0395 (14)	0.0663 (18)	0.0049 (12)	−0.0305 (14)	−0.0033 (12)
C15	0.0390 (15)	0.0451 (15)	0.081 (2)	0.0027 (11)	−0.0110 (15)	−0.0169 (14)
C16	0.0486 (17)	0.0509 (16)	0.0592 (17)	−0.0033 (12)	0.0038 (14)	−0.0092 (13)
C17	0.0487 (16)	0.0398 (14)	0.0388 (13)	−0.0036 (11)	−0.0046 (12)	0.0002 (10)
C18	0.0429 (14)	0.0274 (12)	0.0313 (11)	−0.0033 (9)	−0.0056 (10)	0.0025 (9)
C19	0.0354 (12)	0.0270 (11)	0.0321 (11)	−0.0044 (9)	−0.0034 (9)	0.0033 (9)
C20	0.0521 (15)	0.0367 (13)	0.0352 (12)	−0.0093 (11)	−0.0127 (11)	0.0065 (10)
C21	0.0556 (16)	0.0444 (15)	0.0423 (14)	−0.0065 (12)	−0.0158 (12)	0.0130 (12)
C22	0.0564 (17)	0.0329 (14)	0.0504 (15)	−0.0003 (11)	−0.0097 (13)	0.0121 (11)
C23	0.0701 (19)	0.0294 (13)	0.0453 (14)	−0.0085 (11)	−0.0115 (13)	0.0023 (11)
C24	0.0535 (16)	0.0344 (13)	0.0352 (12)	−0.0054 (11)	−0.0066 (11)	0.0025 (10)
C25	0.0532 (16)	0.0343 (13)	0.0450 (13)	−0.0036 (11)	−0.0219 (12)	0.0005 (10)

### Geometric parameters (Å, °)

**Table:**

S1—C1	1.675 (2)	C10—H10	0.9500
Cl1—C20	1.742 (2)	C11—H11	0.9500
O1—C5	1.218 (2)	C12—C13	1.394 (3)
N1—C1	1.360 (2)	C12—C17	1.396 (3)
N1—N2	1.389 (2)	C13—C14	1.390 (3)
N1—C3	1.476 (2)	C13—H13	0.9500
N2—C2	1.311 (2)	C14—C15	1.381 (3)
N3—C2	1.381 (2)	C14—H14	0.9500
N3—C1	1.397 (2)	C15—C16	1.380 (3)
N3—N4	1.398 (2)	C15—H15	0.9500
N4—C18	1.266 (2)	C16—C17	1.389 (3)
C2—C25	1.485 (3)	C16—H16	0.9500
C3—C4	1.523 (3)	C17—H17	0.9500
C3—C12	1.528 (3)	C18—C19	1.474 (3)
C3—H3	1.0000	C18—H18	0.9500
C4—C5	1.524 (3)	C19—C20	1.399 (3)
C4—H4A	0.9900	C19—C24	1.401 (3)
C4—H4B	0.9900	C20—C21	1.392 (3)
C5—C6	1.496 (3)	C21—C22	1.373 (3)
C6—C11	1.396 (3)	C21—H21	0.9500
C6—C7	1.407 (3)	C22—C23	1.381 (3)
C7—C8	1.383 (3)	C22—H22	0.9500
C7—H7	0.9500	C23—C24	1.383 (3)
C8—C9	1.385 (3)	C23—H23	0.9500
C8—H8	0.9500	C24—H24	0.9500
C9—C10	1.385 (3)	C25—H25A	0.9800
C9—H9	0.9500	C25—H25B	0.9800
C10—C11	1.381 (3)	C25—H25C	0.9800
			
C1—N1—N2	114.03 (16)	C6—C11—H11	119.6
C1—N1—C3	127.05 (17)	C13—C12—C17	118.7 (2)
N2—N1—C3	118.91 (15)	C13—C12—C3	123.07 (19)
C2—N2—N1	104.23 (16)	C17—C12—C3	118.17 (18)
C2—N3—C1	109.24 (16)	C14—C13—C12	120.0 (2)
C2—N3—N4	117.55 (17)	C14—C13—H13	120.0
C1—N3—N4	133.00 (17)	C12—C13—H13	120.0
C18—N4—N3	120.21 (18)	C15—C14—C13	120.8 (2)
N1—C1—N3	101.84 (17)	C15—C14—H14	119.6
N1—C1—S1	127.71 (16)	C13—C14—H14	119.6
N3—C1—S1	130.42 (15)	C16—C15—C14	119.7 (2)
N2—C2—N3	110.66 (18)	C16—C15—H15	120.2
N2—C2—C25	126.39 (19)	C14—C15—H15	120.2
N3—C2—C25	122.95 (18)	C15—C16—C17	120.1 (2)
N1—C3—C4	108.79 (16)	C15—C16—H16	119.9
N1—C3—C12	108.60 (17)	C17—C16—H16	119.9
C4—C3—C12	115.85 (17)	C16—C17—C12	120.7 (2)
N1—C3—H3	107.8	C16—C17—H17	119.7
C4—C3—H3	107.8	C12—C17—H17	119.7
C12—C3—H3	107.8	N4—C18—C19	117.7 (2)
C3—C4—C5	112.93 (17)	N4—C18—H18	121.1
C3—C4—H4A	109.0	C19—C18—H18	121.1
C5—C4—H4A	109.0	C20—C19—C24	117.85 (19)
C3—C4—H4B	109.0	C20—C19—C18	121.47 (19)
C5—C4—H4B	109.0	C24—C19—C18	120.7 (2)
H4A—C4—H4B	107.8	C21—C20—C19	121.0 (2)
O1—C5—C6	121.09 (19)	C21—C20—Cl1	118.29 (18)
O1—C5—C4	120.94 (19)	C19—C20—Cl1	120.74 (16)
C6—C5—C4	117.96 (18)	C22—C21—C20	119.7 (2)
C11—C6—C7	118.5 (2)	C22—C21—H21	120.1
C11—C6—C5	122.84 (19)	C20—C21—H21	120.1
C7—C6—C5	118.63 (19)	C21—C22—C23	120.5 (2)
C8—C7—C6	120.5 (2)	C21—C22—H22	119.8
C8—C7—H7	119.8	C23—C22—H22	119.8
C6—C7—H7	119.8	C22—C23—C24	120.0 (2)
C7—C8—C9	119.9 (2)	C22—C23—H23	120.0
C7—C8—H8	120.0	C24—C23—H23	120.0
C9—C8—H8	120.0	C23—C24—C19	120.9 (2)
C10—C9—C8	120.3 (2)	C23—C24—H24	119.6
C10—C9—H9	119.8	C19—C24—H24	119.6
C8—C9—H9	119.8	C2—C25—H25A	109.5
C11—C10—C9	120.0 (2)	C2—C25—H25B	109.5
C11—C10—H10	120.0	H25A—C25—H25B	109.5
C9—C10—H10	120.0	C2—C25—H25C	109.5
C10—C11—C6	120.7 (2)	H25A—C25—H25C	109.5
C10—C11—H11	119.6	H25B—C25—H25C	109.5
			
C1—N1—N2—C2	0.1 (2)	C6—C7—C8—C9	1.1 (3)
C3—N1—N2—C2	178.94 (17)	C7—C8—C9—C10	−0.4 (3)
C2—N3—N4—C18	173.88 (19)	C8—C9—C10—C11	−0.5 (3)
C1—N3—N4—C18	−12.1 (3)	C9—C10—C11—C6	0.6 (3)
N2—N1—C1—N3	0.1 (2)	C7—C6—C11—C10	0.1 (3)
C3—N1—C1—N3	−178.57 (17)	C5—C6—C11—C10	−179.6 (2)
N2—N1—C1—S1	−177.92 (15)	N1—C3—C12—C13	110.5 (2)
C3—N1—C1—S1	3.4 (3)	C4—C3—C12—C13	−12.2 (3)
C2—N3—C1—N1	−0.3 (2)	N1—C3—C12—C17	−67.4 (2)
N4—N3—C1—N1	−174.73 (19)	C4—C3—C12—C17	169.86 (18)
C2—N3—C1—S1	177.65 (17)	C17—C12—C13—C14	0.3 (3)
N4—N3—C1—S1	3.3 (3)	C3—C12—C13—C14	−177.7 (2)
N1—N2—C2—N3	−0.3 (2)	C12—C13—C14—C15	0.7 (4)
N1—N2—C2—C25	−179.7 (2)	C13—C14—C15—C16	−0.9 (4)
C1—N3—C2—N2	0.4 (2)	C14—C15—C16—C17	0.2 (4)
N4—N3—C2—N2	175.82 (17)	C15—C16—C17—C12	0.7 (4)
C1—N3—C2—C25	179.84 (19)	C13—C12—C17—C16	−0.9 (3)
N4—N3—C2—C25	−4.8 (3)	C3—C12—C17—C16	177.1 (2)
C1—N1—C3—C4	−123.5 (2)	N3—N4—C18—C19	−179.87 (17)
N2—N1—C3—C4	57.9 (2)	N4—C18—C19—C20	−173.6 (2)
C1—N1—C3—C12	109.6 (2)	N4—C18—C19—C24	7.7 (3)
N2—N1—C3—C12	−69.1 (2)	C24—C19—C20—C21	−1.0 (3)
N1—C3—C4—C5	166.45 (17)	C18—C19—C20—C21	−179.8 (2)
C12—C3—C4—C5	−70.9 (2)	C24—C19—C20—Cl1	179.18 (17)
C3—C4—C5—O1	−5.8 (3)	C18—C19—C20—Cl1	0.4 (3)
C3—C4—C5—C6	175.37 (18)	C19—C20—C21—C22	0.4 (4)
O1—C5—C6—C11	179.3 (2)	Cl1—C20—C21—C22	−179.82 (19)
C4—C5—C6—C11	−1.8 (3)	C20—C21—C22—C23	0.6 (4)
O1—C5—C6—C7	−0.3 (3)	C21—C22—C23—C24	−0.9 (4)
C4—C5—C6—C7	178.57 (19)	C22—C23—C24—C19	0.2 (4)
C11—C6—C7—C8	−0.9 (3)	C20—C19—C24—C23	0.7 (3)
C5—C6—C7—C8	178.70 (19)	C18—C19—C24—C23	179.5 (2)
